# Molecular identification of *Pseudodiscus collinsi* from wild Indian elephant (*Elephas maximus indicus*) based on ITS-2

**DOI:** 10.2478/helm-2025-0029

**Published:** 2025-11-26

**Authors:** A. Anaswara, S. Arun, R. Geethu, N. C. Sreenidhi, S. Suriya, S. Harshit, P. K. Binoy, V. Anju, C. K. Deepa, K. G. Ajith Kumar, R. Ravindran

**Affiliations:** 1 Department of Veterinary Parasitology, College of Veterinary and Animal Sciences, Pookode, Kerala Veterinary and Animal Sciences University, Wayanad, Kerala 673 576, India; 2 Assistant Forest Veterinary Offi cer, Kozhikode Forest Division India reghu@kvasu.ac.in

**Keywords:** Amphistomes, Digenetic trematode, *Pseudodiscus collinsi*, Indian elephant, ITS-2

## Abstract

Domestic and wild animals can contract amphistomosis, a disease caused by digenetic trematodes belonging to the superfamily Paramphistomoidea. The importance of these flukes is underestimated worldwide due to their ubiquity and abundance among hosts. *Pseudodiscus collinsi* is a member of the family Paramphistomatidae that infects the colon of equines and elephants. In the present study, the flukes were recovered from the colon of a dead wild Indian elephant (*Elephas maximus indicus*). The flukes were stained using acetyl alum carmine and morphologically identified as *P. collinsi* based on the presence of oral pouches and position of the testes. The polymerase chain reaction (PCR) for amplification of the internal transcribed spacer 2 (ITS-2) region and sequence analysis were performed. The phylogenetic analysis using the Maximum Likelihood (ML) method, based on the Kimura 2-parameter model, revealed the separation of *P. collinsi* (elephant) as a distinct species from the other amphistomes of different hosts. This is the first molecular marker of *P. collinsi* to be presented.

## Introduction

The Asian elephant is one of the few remaining mega-herbivores that have not yet become extinct (Owen-Smith, 1988) and is listed as endangered (EN) because of the reduction in population size (inferred to be at least 50 % over the last three generations) (Williams *et al*., 2020). The largest terrestrial mammal, elephants are susceptible to several ailments analogous to those of horses and cattle. One of the most common illnesses affecting elephants is gastrointestinal parasitism, which invariably impacts their health status and can cause disease and death.

*Fasciola jacksoni* (bile ducts and duodenum), *Pseudodiscus collinsi* (large intestine), *P. hawkesii* (large intestine), *Pfenderius pappillatus* (large intestine), *P. birmanicus* (large intestine), *P. heterocaeca* (large intestine), *Gastrodiscus secundus* (large intestine) and *Bivitellobilharzia nairi* (Portal and mesenteric vein) are the common flukes found in elephants (Fowler & Mikota, 2006; Bapu, 1936). The members of the Paramphistomoidea superfamily are widely distributed across the world, with the highest prevalence in the tropical and subtropical regions (Tandon *et al*., 2014). Their immature flukes are embedded in the intestinal mucosa and cause hemorrhagic or catarrhal enteritis. The adults collectively cause a disease referred to as amphistomosis or stomach fluke disease in mammalian livestock. *Pseudodiscus collinsi is one of the members of the superfamily Paramphistomoidea (Cobbold, 1882)* that occurs in the colon of elephants and equines. *Pseudodiscus* infection in elephants is characterized by clinical symptoms like anorexia, dehydration, inability to walk and severe foul-smelling diarrhea (Muraleedharan & Keshavaprasad, 2001). *Pseudodiscus collinsi* and *Gastrodiscus secundus* were previously reported in two captive Indian elephants with edema, pinhead-sized hemorrhages, and ulcerative patches in their caecal mucosa (Islam *et al*., 1999). Amphistomosis is commonly diagnosed by detecting eggs in feces and morphologically identifying adult flukes. The species-level identification of amphistomes based on ova is a big challenge due to their similarity. Histological examination of the median sagittal section of the acetabulum, pharynx and genital atrium of flukes is another mode of morphological identification (Nasmark, 1937). The previous reports of *P. collinsi* (Bhalerao 1931, 1933; Nasmark, 1937; Malik *et al*., 1958; Rai, 1959; Peter & Srivastava, 1960; Gupta & Walia,1970; Muraleedharan & Keshavaprasad, 2001) were based on the adult morphology, without genetic confirmation of species identity. Molecular characterization might help in identifying and studying genetic variation and evolutionary history. In the present study, adult flukes from the large intestine of a wild Indian elephant were morphologically described and molecularly characterized by the ribosomal ITS-2 sequence.

## Materials and Methods

### Sample collection and morphological identification

The flukes were collected from the colon of a dead adult wild Indian elephant (*Elephas maximus indicus*) during the postmortem at Naikkatty, Wayanad Wildlife Division, Sulthan Bathery, Kerala, India, on 3 July 2023. The flukes were brought to the Department of Veterinary Parasitology, College of Veterinary and Animal Sciences, Pookode, Wayanad. They were washed in normal saline (0.9 %), stained using acetyl alum carmine stain, cleared in creosote and mounted using dibutylphthalate polystyrene xylene (DPX) (Soulsby, 1982; Singh, 2003). They were examined under the stereozoom microscope (Leica M 205C, Germany). The morphological identification of the flukes was performed using the monograph of the helminth parasites of elephants (Westhuysen, 1938). The remaining flukes were stored at -20 ° C for DNA isolation and further processing. The measurements (mean±SD) of the flattened stained flukes were determined.

*Genomic DNA extraction and polymerase chain reaction (PCR)* The genomic DNA was extracted from three adult flukes by the DNeasy® blood and tissue kit (M/s. Qiagen, Hilden, Germany) according to the manufacturer’s protocol. The fluke DNA samples were quantified using a Nanodrop 2000C spectrophotometer (M/s. Thermo Scientific, Massachusetts, USA). The DNA was then stored at -20 ° C until further procedures. The genomic DNA was used for amplification of the internal transcribed spacer 2 (ITS-2) region (Anderson & Barker, 1998; Luton *et al*., 1992) (Table 1). Polymerase chain reaction (PCR) cycles were performed in an automated thermal cycler with a heated lid (Eppendorf, Germany). The PCRs were set up in a total volume of 25 μL using 10X buffer (Thermo Scientific, USA), dNTP mix (0.2 mM, Thermo Scientific, USA), 0.5 U *Taq* DNA Polymerase (Thermo Scientific, USA), 10 pmol each of forward and reverse primers, 50 – 100 ng of template DNA and nuclease-free water.

**Table 1.** Primer details and cycling conditions.

Gene	Primer name	Sequence	No of base pairs (bp)	Product size (bp)	Cycling conditions	References
**ITS2**	Forward primer GA1	5′-AGA ACA TCG ACA TCT TGA AC-3′	20	~400 bp	$\begin{array}{l} \text{ Denaturation: }95^{{}^{\circ}}\text{C}\;\text{-}\;1\;\text{min}\\ \text{ Annealing: }55^{{}^{\circ}}\text{C}\;\text{-}\;2\;\text{min}\\ \text{ Extension: }74^{{}^{\circ}}\text{C}\;\text{-}\;1\;\text{min}30\text{sec} \end{array}\}\;1\text{ cycle }$	Anderson and Barker, 1998; Luton *et al*., 1992
	Reverse primer BD2	5′-TAT GCT TAA ATT CAG CGG GT-3′	20		$\begin{array}{l} \text{ Denaturation: }95^{{}^{\circ}}\text{C}\;\text{-}\;30\text{sec}\\ \text{ Annealing: }55^{{}^{\circ}}\text{C}\;\text{-}\;30\text{sec}\\ \text{ Extension: }74^{{}^{\circ}}\text{C}\;\text{-}\;1\;\text{min}30\text{sec}\\ \text{ Final extension: }74^{{}^{\circ}}7\;\;\text{-}\;\text{min} \end{array}\}30\text{ cycles }$	

### Agarose gel electrophoresis and gel documentation

The PCR-amplified sample was analyzed by agarose gel electrophoresis using 1 % agarose (M/s. Sigma Aldrich, USA) in 0.5x Tris borate EDTA (TBE) (pH 8.0; 0.045 M Tris, 0.045 M boric acid, 1 mM EDTA) and with 0.00005 % (0.5μg/ML) ethidium bromide for DNA staining. PCR product (5μL) was mixed with 6X gel loading dye (M/s. Thermo Scientific, USA) in a 5:1 ratio and applied to each well. The 100 bp DNA ladder from Invitrogen (M/s. Thermo Fisher Scientific, Lithuania) (3 μL) was used as a molecular marker. Gels were run in 0.5x TBE buffer for 75 min at 80V, 53 mA power supply. The PCR products were visualized and photographed on a gel documentation system (UVITECH, Cambridge).

### Phylogenetic analysis

The amplicons of PCR targeting ITS-2 were sent for sequencing in both forward and reverse directions at M/s. AgriGenome Labs Private Ltd, Cochin, Kerala. The nucleotide sequences were verified and edited using the BioEdit program. They were analyzed for their identity using the National Center for Biotechnology Information-Basic Local Alignment Search Tool (NCBI-BLAST) (www.ncbi.nlm.nih.gov/BLAST). Using the ClustalW program, multiple sequence alignment was performed using the previously published sequences from GenBank. The aligned sequences were trimmed to the same length (with gaps) from which a phylogenetic tree was constructed based on the Maximum Likelihood (ML) method, using the MEGA 11 program with the suitable models. The reliability of the topologies was tested by bootstrapping with 1000 replications [ITS-2, Kimura 2-parameter model].

## Ethical Approval and/or Informed Consent

This article does not contain any studies with human participants or animals.

## Results

**Fig. 1.**
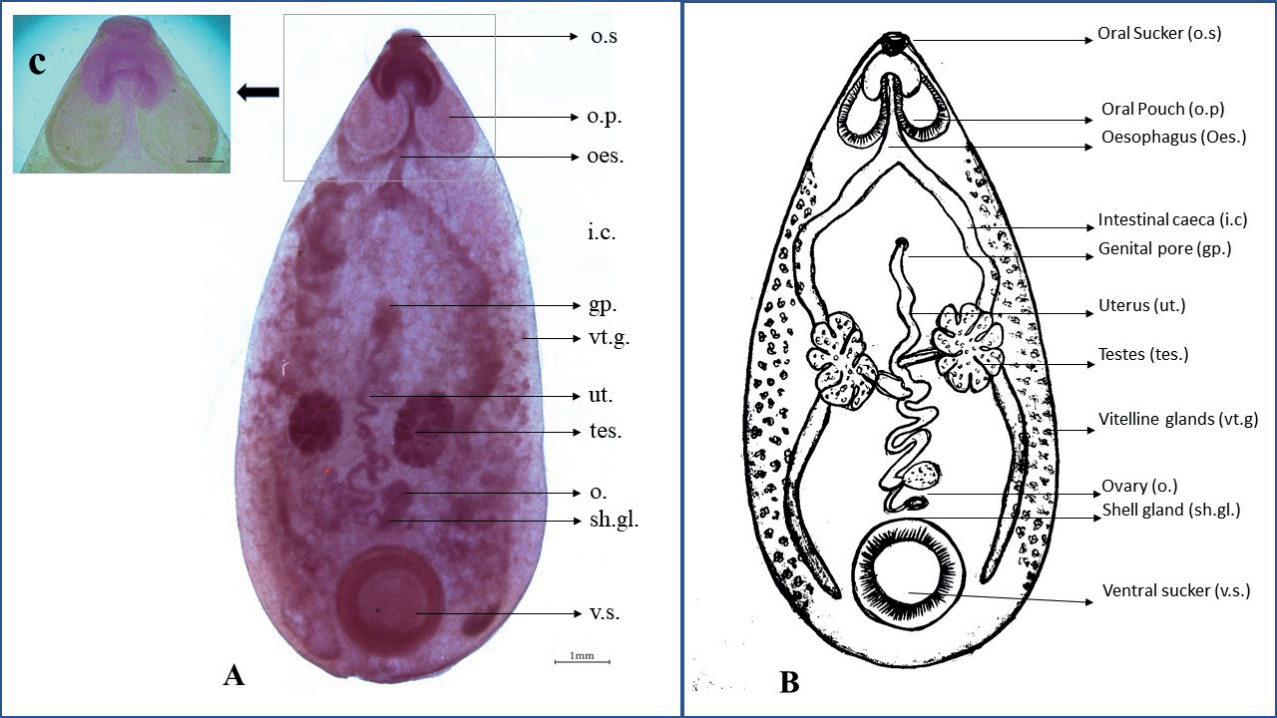
*Pseudodiscus collinsi*: A. Acetyl alum carmine stained adult intestinal fluke (o.s.: Oral sucker; o.p.: Oral pouch; oes.: Oesophagus; i.c.: Intestinal caeca; gp.: Genital pore; vt.g.: Vitelline glands; ut.: Uterus; tes.: Testes; o.: Ovary; sh.gl.: Shell gland; v.s.: Ventral sucker) B. Line diagram of adult *Pseudodiscus collinsi* C. Oral pouches of adult *Pseudodiscus collinsi*.

The collected flukes were oval and pinkish in color. The anterior extremity was bluntly pointed, and the posterior extremity was round, almost semi-circular, and the lateral margin was convex. The adult fluke measured approximately 10.10 mm – 13.91 mm (12.00 ± 1.16, n=22) in length and had a maximum width of 4.12 mm – 6.51 mm (5.72 ± 0.79, n=22) (Fig.1).

The mouth was surrounded by an oral sucker, which showed bilobed oral pouches 1.36 mm – 1.60 mm (1.53 ± 0.07, n=22) long and 0.76 mm – 0.91 mm (0.86 ± 0.06, n=22) wide (Fig. 1C). Each lobe communicated posteriorly with a diverticulum. Esophagus originates from the portion between the two oral pouches, 1.52 mm – 1.75 mm (1.61 ± 0.08, n=22) mm long, bulb absent; branches into two intestinal caeca. The intestinal caeca were wavy, ran posteriorly and reached up to the middle of the posterior sucker, 16.56 mm – 19.02 mm (17.27 ± 0.93, n=22) long. Each intestinal cecum showed an inward bend at the level of the testes. Two large, deeply lobed testes were situated side by side, slightly posterior to the center of the body, 1.72 mm – 2.05 mm (1.93 ± 0.07, n=22) long and 1.36 mm – 1.76 mm (1.51 ± 0.10, n=22) wide. The ovary was observed in the median line and in between the testes and posterior sucker, 0.51 mm – 0.72 mm (0.66 ± 0.09, n=22) long and 0.50 mm – 0.60 mm (0.57 ± 0.06, n=22). The uterus was coiled and ran anteriorly in between the testes along the dorsal surface. The ventral suckers are 2.01 mm – 2.41 mm (2.21 ± 0.11, n=22) long and 1.67 mm – 2.20 mm (1.90 ± 0.13, n=22). The vitelline glands showed numerous follicles, scattered on the outer side of the intestinal caeca up to their posterior end (Fig. 1A&B).

**Fig. 2.**
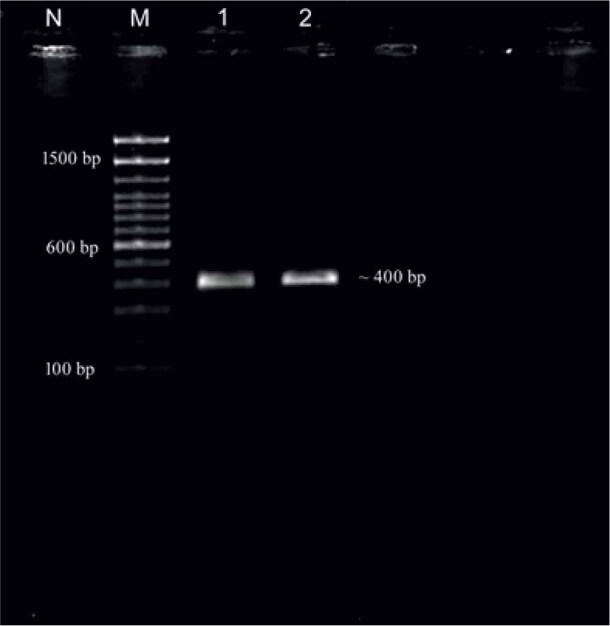
PCR amplification ITS-2 (~400 bp) of *P. collinsi* (Lane M: 100 bp plus ladder, Lane 1,2: *P. collinsi* samples, Lane N: Negative control).

**Fig. 3.**
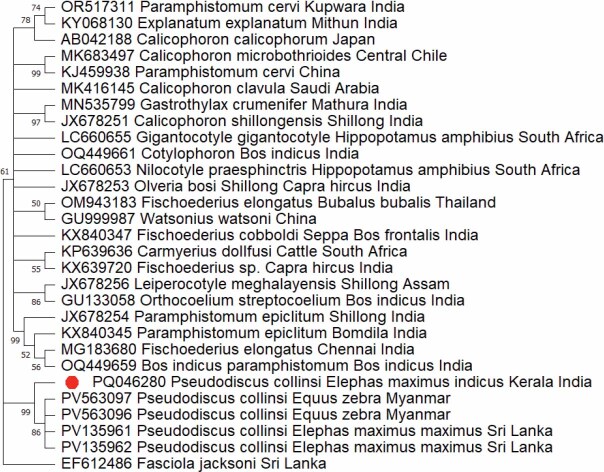
Phylogenetic analysis based on ITS-2 of *P. collinsi*. The evolutionary history was inferred using the Maximum Likelihood (ML) method. The optimal tree is shown. The percentage of replicate trees in which the associated taxa clustered together in the bootstrap test (1000 replicates) are shown next to the branches. The evolutionary distances were computed using the Kimura 2-parameter method and are in the units of the number of base substitutions per site. The rate variation among sites was modelled with a gamma distribution (shape parameter = 1). This analysis involved 25 nucleotide sequences. All ambiguous positions were removed for each sequence pair (pairwise deletion option). There was a total of 335 positions in the final dataset. Evolutionary analyses were conducted in MEGA 11 (Tamura *et al*., 2021).

The PCR targeting the ITS-2 region amplified a ~400 bp product (Fig. 2). The sequences of all three flukes were identical, hence we submitted a single representative sequence to GenBank. The aligned sequences (NCBI accession number PQ046280) showed maximum similarity with *Calicophoron microbothrioides* (94.77 %). There is no data for *P. collinsi* available in the GenBank database. The phylogenetic analysis indicated that the *P. collinsi* from the elephant in the present study distinguished itself from all other isolates of amphistomes from different species of animals (Fig. 3).

## Discussion

The flukes were identified as *P. collinsi* based on the morphological characters, mainly the oral pouches and the arrangement of testes (Westhuysen, 1938). The genus *Pseudodiscus* has two different species, *P. collinsi* and *P. hawkesii. Pseudodiscus collinsi* showed oral pouches, intestinal caeca reaching up to the middle of the posterior sucker, deeply lobed side-by-side testes and an ovary situated in the midline between the testes and the posterior sucker. *Pseudodiscus hawkesii* is more elongated than *P. collinsi* with an acutely pointed oral pole, the intestinal caeca end in the equatorial zone of the posterior sucker, and the testes are deeply lobed, placed one behind the other (Westhuysen, 1938).

*Pseudodiscus collinsi* was morphologically identified in several studies conducted on Indian elephants (Bhalerao 1931, 1933; Malik *et al*., 1958; Rai, 1959; Muraleedharan & Keshavaprasad, 2001). Peter and Srivastava (1960) described redia, cercaria, metacercaria and adult flukes of *P. collinsi*. Gupta and Walia (1970) described and illustrated the gross anatomy and histology of *P. collinsi*. Nasmark (1937) used the histological examination method to identify *P. collinsi*. Islam *et al*. (1999) studied the pathology of concurrent *P. collinsi* and *Gastrodiscus secundus* infections in captive Indian elephants, resulting in edema, pin-head-sized hemorrhages, and ulcerative patches in the caecal mucosa.

The evolutionary analysis using ITS-2 revealed that the *P. collinsi* from wild Indian elephant stood separated from all other species. The lack of sufficient numbers of sequences for *P. collinsi* ITS-2 in GenBank was a constraint during phylogenetic analysis. The present study formed the first sequencing of the ITS-2 molecular marker of *P. collinsi*.

The impact of flukes in the large intestine of wild elephants was less understood. There is an urgent need to re-examine these flukes to understand how they interact with and impact their host. In addition, the results of this study can be used to understand the relationship between the host and parasite, including the life cycle, pathology, ecology and epidemiology.

## Conclusion

*Pseudodiscus collinsi* flukes recovered from the cecum of the wild Indian elephant were characterized based on both morphological features and ribosomal markers. The phylogenetic analysis of ITS-2 sequence revealed a unique branch for the species distant from other species but a close association in the Paramphistomoidea superfamily. This study represented the first molecular characterisation of *P. collinsi* from wild Indian elephants using the nuclear ITS-2.
